# Neuroendocrine tumours through an epigenetic lens: Emerging insights for diagnosis and treatment

**DOI:** 10.1111/jne.70211

**Published:** 2026-06-07

**Authors:** Victoria Jacquot, Benjamin Chevalier, Thomas Walter, Benjamin Gibert, Maria Ouzounova

**Affiliations:** ^1^ Gastroenterology and Technologies for Health, Centre de Recherche en Cancérologie de Lyon, INSERM U1052‐CNRS UMR5286, Centre Léon Bérard Université Claude Bernard Lyon1 Lyon France; ^2^ Hospices Civils de Lyon, Hôpital Edouard Herriot Service de Gastroentérologie et d'Oncologie Digestive Lyon France

**Keywords:** DNA methylation, epigenetic‐targeting drugs, neuroendocrine tumors, non‐coding RNAs, histone modifications

## Abstract

Neuroendocrine tumours (NETs) are well‐differentiated epithelial neuroendocrine neoplasms that frequently develop in the small intestine, pancreas, and lungs. NETs originate from neuroendocrine cells specialized in hormone secretion implicated in a number of physiological processes. Their malignant transformation is characterized by low mutational burden, suggesting that epigenetic mechanisms may be at play. Recent understanding of epigenetic events driving cancer cell plasticity and tumour initiation has led to advances in the diagnosis and prognosis of NETs. Here, we provide a brief overview of NETs, including their current diagnosis and management, and present recent progress in understanding the role of epigenetic regulation, highlighting how this may influence NET tumorigenesis and may be used in therapeutic applications. Finally, this literature review emphasizes the need to gather more data on these rare malignancies to improve patient outcome.

## 1 INTRODUCTION

Neuroendocrine tumours (NETs) develop from neuroendocrine cells, which are present in all epithelial tissues. These neuroendocrine cells secrete hormones and neurotransmitters into the surrounding tissues/bloodstream in response to neuronal or chemical signals. NETs most commonly develop in the small intestine (siNETs), the pancreas (pNETs) and the lungs.^1^ Their molecular landscape has been widely characterized over the past 15 years, revealing heterogeneity between locations. Indeed, whereas alterations in *MEN1*, *DAXX/ATRX*, and genes involved in the mTOR pathway are frequent in pancreatic NETs, there are no recurrent gene alterations observed in siNETs except *CDKN1B* mutation in 8.5% of siNETs patients^2^ and only *MEN1*, *ARID1A*, and *EIF1AX* in less than 10% of lung NETs.^3^, ^4^, ^5^, ^6^ Thus, aside from *MEN1*, there is no strong driver mutation associated with NET tumorigenesis. Hence, we believe that additional mechanisms are implicated in NET development, likely linked to epigenetic processes.

Such epigenetic mechanisms have been reported in the tumorigenesis of various cancers, notably in association with cellular plasticity.^7^ Among the most studied epigenetic modifications are DNA methylation, histone modifications, and non‐coding RNAs. Aside from shedding light on fundamental processes during tumorigenesis, studying epigenetic modifications is also of clinical interest. Indeed, epigenetic modifications are by definition reversible and represent an attractive targeting strategy through the development of epigenetic inhibitors, several of which are already routinely used.^8^

In this review, we will first synthesize the current state of knowledge of NET biology. We will then examine recent advances in the epigenetic landscape of neuroendocrine neoplasia, with a focus on epigenetic mechanisms implicated in tumorigenesis, their prognostic relevance for patient survival, and their emerging diagnostic and therapeutic utility.

## 2 NEUROENDOCRINE TUMOURS

### 2.1 General features of neuroendocrine tumours

Neuroendocrine neoplasia (NENs) are epithelial neoplasms arising from neuroendocrine cells of the diffuse endocrine system.^9^ According to the WHO 2022 Classification of Endocrine and Neuroendocrine Tumours, NENs are divided into well‐differentiated neuroendocrine tumours (NETs) and poorly differentiated neuroendocrine carcinomas (NECs). NETs are classified as low‐grade (G1), intermediate (G2), and high‐grade (G3) based on proliferation markers (mitotic count and Ki67).^5^, ^10^ In contrast, NECs are characterized by poor epithelial differentiation, marked cellular atypia, and frequent molecular or genetic abnormalities, while retaining expression of neuroendocrine markers.^11^

NETs are either functional, in that they exert symptoms through neurotransmitters and hormone secretion (mainly gastrin, serotonin or insulin) which can be life‐threatening or non‐functional, the latter representing >80% of patients.^12^ NETs also display a marked heterogeneity in their incidence, metastatic patterns, and prognosis, largely due to their site of origin. Furthermore, their tumour immune microenvironment is considered ‘cold’, with low immune cell infiltration, reflected by poor response to immunotherapy of patients harbouring NETs.

Risk factors associated with the formation of NETs include age and genetic predisposition, such as germinal mutations in *MEN1*, *VHL*, *CDKN1B* or *TSC*.^13^, ^14^, ^15^ Loss of heterozygosity and loss‐of‐function of the epigenetic regulators and tumour suppressor genes MEN1 and DAXX are key events facilitating the development of NETs in hormone‐releasing tissues.^16^, ^17^ Indeed, more than 40% of patients with sporadic NETs, including insulinomas, as well as 20%–40% of patients with gastroenteropancreatic NETs (GEP‐NETs), exhibit a heterozygous loss of *MEN1*.

The incidence rate of NETs in the United States is around 8.2 per 100,000 people per year and from 1 to 7 per 100,000 people per year in the world and has been increasing over the past two decades, likely due to a better understanding of the disease and improved diagnosis.^14^, ^18^ At the metastatic stage, the main locations of NETs are the small intestine (si), the pancreas (p), and the lungs.

### 2.2 Small intestine neuroendocrine tumours (siNETs)

The most frequent NETs are siNETs that include tumours located in the duodenum, jejunum or ileum.^19^ The incidence of siNETs has continually increased over the past decades, with rates being multiplied by 6.4 in the United States between 1973 (1.09 per 100,000) and 2012 (6.98 per 100,000).^1^, ^20^ Though the average age at diagnosis of primary NETs ranges from 47 to 68 years, siNETs diagnosis is generally made around 66–67 years, with median 5‐year and 10‐year overall survival (OS) rates of 67% and 37%, respectively.^19^, ^20^, ^21^ Survival rates decrease drastically with siNET expansion from ~160 months in patients with localized disease to ~60 months in metastatic patients.^10^ Early stages of NET development are typically asymptomatic, making diagnosis particularly challenging, especially in siNETs.^22^ Patients are thus diagnosed at advanced stages, after metastatic dissemination, primarily in the mesenteric lymph nodes, as well as in the liver, lungs or peritoneum.^23^

siNETs are associated with serotonin secretion resulting in symptoms known as ‘carcinoid syndrome’ encompassing diarrhoea, flush, wheezing, and bronchospasms in 30%–40% of cases. In 20%–50% of cases, carcinoid syndrome can lead to carcinoid heart disease (CHD), associated with the development of fibrosis in the right heart valves.^24^

### 2.3 Pancreatic neuroendocrine tumours (pNETs)

As for siNETs, the incidence of pNETs has gradually increased between 1973 (~0.2 per 100,000) and 2012 (~0.8 per 100,000).^17^ The OS rates of patients with pNETs are slightly higher than those with siNETS with median 5‐year and 10‐year OS of ~75% and 60%, respectively.^19^, ^20^ Similarly to siNETS, survival declines rapidly with tumour expansion from ~240 months in patients with localized disease to ~25 months in metastatic patients.^10^

In addition, pNETs may also be non‐functional or functional, the latter including but not limited to insulinoma, in which excessive insulin is secreted, leading if repeated to potentially life‐threatening hypoglycaemia, or gastrinoma in which excessive gastrin is secreted and can lead to diarrhoea and stomach or duodenal ulcer.^10^

### 2.4 Lung neuroendocrine tumours

Lung NETs may occasionally be associated with serotonin secretion and lead to carcinoid syndrome. In terms of incidence, this has risen from 0.3 per 100,000 in 1973 to 1.6 per 100,000 in 2012.^17^ The median 5‐year and 10‐year OS rates of patients harbouring lung NETs are 50% and 40%, respectively. Moreover, this OS drops from ~240 months in patients with localized disease to ~10 months in metastatic patients.^25^

### 2.5 Diagnosis and management

Techniques used to diagnose NETs include standard imaging, such as computed tomography (CT) scans and magnetic resonance imaging (MRI), to determine the exact location and nature of the tumour, as well as the potential presence of distant metastases.^26^ The use of positron emission tomography/scanner (PET‐CT) radiopharmaceuticals, such as ^68^Ga‐DOTATOC, which targets the somatostatin receptor expressed by NETs, or ^18^FDG in case of an aggressive disease (NET G3 or NEC), provides a more accurate image.^27^ The former is particularly more sensitive at detecting the presence of metabolically active metastases and micrometastases.^27^, ^28^ Routine analyses like tissue biopsy, endoscopy, and endoscopic ultrasound (EUS) can also be used for diagnosis and/or for patient monitoring. Histological analysis can then be conducted using biological markers, such as the proliferation marker Ki‐67 and NET‐specific markers chromogranin A (CgA), synaptophysin (Syn), and somatostatin receptor type 2 (SSTR2A).^29^ Additionally, the serotonin degradation product, 5‐hydroxyindoleacetic acid (5‐HIAA) can be studied in both urine and serum samples.^28^, ^30^, ^31^

Among the treatments available, surgical resection is performed as a first‐line approach to curatively control disease progression in the case of localized disease or locoregional lymph node involvement. For metastatic NETs, treatment choice is made by expert centres based on several parameters, including primary tumour location, differentiation and grade, metastatic sites and burden, tumour slope, and symptoms. For NETs with low tumoral burden, slowly evolving, asymptomatic, and primarily liver metastases, careful monitoring or somatostatin analogues (SSA) are indicated.^32^, ^33^ In the event of high tumoral burden and/or quickly evolving NETs, therapeutic options depend on the primary tumour location, with pNETs, for instance, receiving chemotherapy as first‐line treatment. The choice of regimen can also be guided by MGMT status.^34^ Second‐line treatments and other systemic treatments include radioligand therapy (RLT), mTOR inhibitors, or antiangiogenic drugs.^35^, ^36^, ^37^ Similar approaches are used for patients harbouring lung NETs.^38^ In siNETs, owing to the poor efficacy of chemotherapy, RLT is applied immediately after SSA failure, prior to considering mTOR inhibitors, antiangiogenic agents, or interferon alpha.^39^, ^40^

Interventional radiology, such as embolization or chemoembolization (chemotherapy injection through hepatic artery), can also be considered.^41^ In addition to their anti‐tumoral action, these agents may reduce hormone secretion and associated symptoms.^12^ Supportive care can also be implemented to control hormone secretions in patients with functional tumours, tailored to individual needs.^42^

## 3 EPIGENETIC MODIFICATIONS IN NETs

Descriptions of these rare malignancies are scarce, which poses a significant challenge in order to understand their biology and improve their treatments. Several studies have shown that they are stable tumours with few or no significant driver mutations, particularly in the case of siNETS.^43^ Consistently pancreatic and lung NETs, display very few exonic non‐silent mutations, and even exhibit an overall mutational burden substantially lower than that observed in most epithelial malignancies.^44^ This finding questions the underlying mechanisms leading to tumorigenesis, highlighting dysregulated epigenetic events as potential contributors to NET initiation and progression. Epigenetic mechanisms are reversible and heritable changes in gene expression that are not due to changes in the DNA sequence itself. These mechanisms occur before or during transcription and include DNA methylation, histone modification and non‐coding RNAs.^45^

### 3.1 DNA methylation: Mechanisms, regulation and implications in cancer

**FIGURE 1:**
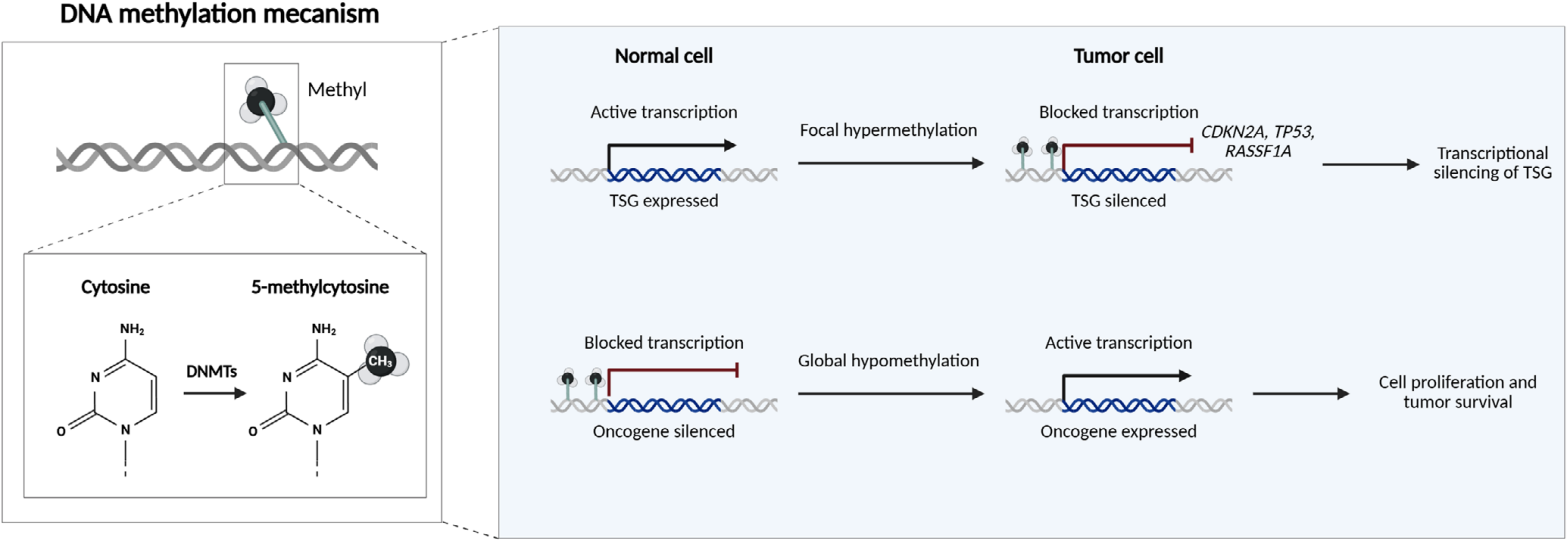
Epigenetic shift from physiological regulation to tumorigenesis in siNETs. DNA methylation mediates transcriptional silencing of tumour suppressor genes and activation of oncogenes, driving tumorigenesis through epigenetic reprogramming.

DNA methylation occurs primarily on cytosines of CpG dinucleotides and involves the addition of a methyl group (CH_3_). This methylation has been extensively described during development, genomic imprinting, transcriptional regulation, genomic stability, and chromatin structure.^46^ Methyl groups are added to DNA by enzymes called methyltransferases (DNMTs), and these groups can be removed by demethylase enzymes, such as TET (Ten‐eleven translocation). These enzymes work in cooperation with CpG island (CGI)‐binding proteins and chromatin remodelling factors.^47^ Approximately 70% of human genes are transcribed from CGI promoters. DNA methylation of cytosines within CGIs leads to transcriptional silencing that must thus be properly regulated.^48^ Alterations in DNA methylation are often associated with various human diseases.^49^ In cancer, abnormal DNA methylation is often correlated with inappropriate repression of tumour suppressor genes, while a lack of methylation on oncogenes can lead to their expression (Figure 1). The loss of DNA methylation at CpG dinucleotides was the first epigenetic abnormality identified in cancer cells.^50^ A global hypomethylation of DNA and targeted hypermethylation of specific genes are also reported in cancer.^46^ Beyond transcriptional repression, abnormal DNA methylation can lead to genetic instability, cell cycle abnormalities, transcription factor dysfunction, or a failure to trigger apoptosis.^51^ For instance, mutations in the *DNMT3B* gene (DNA methyltransferase 3 Beta), which encodes an enzyme responsible for adding methyl groups to cytosines in DNA, affect normal gene expression and play a role in cancer (44). Elevated *DNMT3B* expression has been documented in colorectal, endometrial, and hepatocellular carcinomas; however, the mechanistic contributions of this enzyme to tumour development and progression remain insufficiently characterized.^52^

#### 3.1.1 DNA methylation in siNETs

Several studies have shown that DNA methylation plays a role in NETs, including siNETs. siNETs exhibit a global hypomethylation that leads to the hyperactivation of genes involved in cell proliferation and tumour survival.^53^ In addition, hypermethylation of tumour suppressor gene promoters (sometimes correlated with the presence of metastases), such as *CDKN2A* (= p16/p14), *TP53*, and *RASSF1A*, has been reported, leading to transcriptional silencing of these specific genes^53^, ^54^ (Figure 1).

In addition, recent work on small intestine and appendix NETs has revealed distinct methylation patterns associated with three molecular subtypes (18LOH, MultiCNV, NoCNV). Notably, genes involved in G‐protein coupled receptor (GPCR) signalling (such as SST and TRTH) were differentially methylated, some of which were associated with patient survival. These findings further support the role of DNA methylation as a molecular classifier with potential therapeutic implications for appendix NETs and siNETs.^55^

##### DNA methylation as an epigenetic biomarker in siNETs

Within the landscape of DNA methylation biomarkers in siNETs, patient‐derived studies demonstrate recurrent promoter hypermethylation of candidate genes, which in turn suppresses the expression of tumour‐suppressor and signalling pathways, including *WIF1*, *RASSF1A*, *CTNNB1*, *CXCL14*, *NKX2‐3*, *CDH1*, *P16*, and *LAMA1*. For instance, though *WIF1* methylation was higher in siNET metastases, its expression was lower than primary tumours. It was also reported that low *RASSF1A* and *P16* expression was associated with poor overall survival. Globally, the methylation of LINE1 repeats was reduced in tumours compared to normal tissue, linked to chromosome 18 loss. In vitro, treatment with 5‐azacytidine, a demethylating agent, led to a reduction of DNA methylation and to an increase in the expression of these genes. Further studies regarding *WIF1*, *RASSF1A*, or *P16* are necessary, as they may be prognostic biomarkers of siNETs.^53^

In addition, a 97‐tumour series identified 21 epigenetically‐dysregulated genes including *CDX1*, *CELSR3*, *FBP1* and *GIPR*, with recurrent changes in methylation in 74% to 86% of cases, allowing to classify siNETs into 3 groups after primary tumour resection, correlated with progression‐free survival, supporting their role as prognostic biomarkers.^56^

Finally, methylation of the SST/SST2 promoter is altered in siNETs, as evidenced in vitro and in vivo, but also negatively correlated with SST2 mRNA levels. This finding exposes methylation as a regulator of somatostatin‐receptor expression, which may be relevant for imaging and therapy.^57^, ^58^

In conclusion, defects in DNA methylation are prevalent in siNETs. They generate global and gene‐specific signatures correlated with prognosis and regulate key therapeutic targets, making DNA methylation a rich source of current and emerging biomarkers in siNETs.

#### 3.1.2 DNA methylation in pNETs

Pancreatic NETs are associated with a well‐defined DNA methylation landscape.^59^ Several studies have demonstrated that pNETs exhibit widespread promoter methylation. Notably, hypermethylation of the tumour suppressor gene *RASSF1A* has been reported in up to 80% of pNETs and is associated with an increase in tumour size, aggressiveness, and tendency to metastasise. Moreover, *CDKN2A* (hypermethylated in up to 40% of pNETs) and *TIMP3* (hypermethylated in up to 44% of pNETs) hypermethylation has been correlated with proliferation, metastasis, and poor prognosis.^60^

Mechanistic studies have also linked DNA methylation to tumour progression in pNETs. It was shown that the expression of *NAP1L1* promotes tumour cell proliferation and metastasis by methylating the promoter of the tumour suppressor gene *CDKN1C* (p57KIP2), leading to its transcriptional repression.^61^ In addition, an important protein involved in chromatin structure regulation is DAXX. DAXX‐deficient tumours, like pNETs, exhibit DNA hypomethylation, which is associated with poor patient prognosis.^62^ Thus, highlighting a direct functional connection between aberrant DNA methylation and tumour progression.

Global methylome analyses have also emerged in NETs and especially pNETs. Indeed, each type of NET was reported to exhibit a specific epigenetic signature, allowing a clear distinction between them according to their primary tumour site. MEN1‐related pNETs display more hypermethylated CpGs, while VHL‐related tumours are more hypomethylated. In addition, hypermethylated genes in pNETs are more abundant in pathways involved in DNA repair, chromatin remodelling, telomere maintenance, transcription regulation, cell‐cycle regulation pathways, protein synthesis and processing, indicating a major epigenetic role in tumorigenesis. Furthermore, compared to different NET primary tumours, pNETs display the highest number of hypermethylated CpGs, followed by siNETs and duodenal NETs (DNETs). Interestingly, the APC 1A promoter is hypermethylated specifically in MEN1‐related pNETs. This involvement in MEN1‐related pNET tumorigenesis was not previously described, representing a new epigenetic mechanism in this subtype that may act as an additional oncogenic hit.^44^

##### DNA methylation as an epigenetic biomarker in pNETs

A study in pNETs demonstrated that DNA methylation patterns can delineate patient subgroups with distinct clinical trajectories (tumour behaviour, treatment response, prognosis), paving the way for more tailored therapeutic interventions.^63^, ^64^ Hypermethylation of promoters of tumour suppressor genes could also be used as potential biomarkers, which could orient therapeutic strategies, such as the restoration of normal methylation or reversion of methylation alterations.^65^, ^66^

Moreover, *MEN‐1*‐associated pNETs show widespread CpG hypermethylation compared to VHL‐related and sporadic pNETs, with distinct methylome signatures according to the syndrome and primary site. In addition, *MGMT* hypermethylation is observed in up to 50% of non‐functional pNETs and is associated with response to temozolomide therapy and other alkylating agents. The *MGMT* gene encodes the O6‐methylguanine‐DNA methyltransferase, a DNA repair enzyme that protects cells against damage produced by alkylating agents used in chemotherapy. This methylation status of the *MGMT* promoter may thus serve as a predictive biomarker of response to alkylating agents in *MEN‐1*‐expressing patients with advanced tumours. In addition, the *RASSF1A* tumour‐suppressing gene seems to be an epigenetic marker of *MEN‐1* insulinomas, whereas hypermethylation of the *MGMT* gene promoter was shown to be an epimarker of non‐functional pNETs, suggesting the possibility of specifically targeting both tumours through these epigenetic markers.^67^

Furthermore, LINE‐1 methylation, considered to be a surrogate of global DNA methylation, was proposed as an effective predictor of outcome in pNETs, as hypomethylation is associated with a higher tumour grade.^65^ Recent studies also suggest that methylated DNA markers (MDMs) could assist in pNETs diagnosis and that DNA methylation in pNET tumorigenesis could be linked to histological grade and clinical prognosis.^59^

Overall, these findings highlight changes in DNA methylation in pNETs as potential diagnostic and prognostic biomarkers, as well as strong contributors to enhanced pNET classification. The identification of specific methylation signatures and biomarkers supports the development of more personalized treatment strategies and improved clinical management of pNET patients.

#### 3.1.3 DNA methylation in lung NETs

Compared to other NETs, DNA methylation in lung NETs has been less extensively studied. Recent data from lung NETs highlight DNA methylation as a promising biomarker, in contrast to the more defined pNETs. Aberrant methylation of promoter regions in tumour suppressor genes frequently results in their transcriptional silencing, thereby promoting tumour growth and progression. A well‐known example is the hypermethylation of the *CDKN2A* gene, common to both lung NETs and pNETs, which encodes the p16INK4a protein (2). In addition, *RASSF1A* (common to pNETs) and *SOX17* are frequently methylated in lung NETs (2).

While DNA methylation is a key epigenetic mechanism in NETs, including siNETs, pNETs, and lung NETs, histone modifications represent another major aspect of chromatin regulation influencing gene expression and tumour progression.

##### DNA methylation as an epigenetic biomarker in lung NETs

Methylation profiling in lung NETs has identified distinct epigenetic clusters that align with key biological and clinical features, including histological subtype (typical vs. atypical carcinoids), metastatic potential, endocrine activity, and SSTR2 expression. Functionally, the differentially methylated genes are predominantly associated with cell‐signalling pathways. These findings suggest that DNA methylation patterns could enhance the diagnostic stratification of lung NETs.^68^

Furthermore, in lung NETs and NECs (including small cell lung cancer cells or SCLC), hypermethylation of the MGMT promoter is detected in a sizable fraction of patients and may act as a predictive marker of temozolomide treatment response. Although some studies report limited association between MGMT promoter methylation and survival outcome, clinical guidelines recommend the use of this MGMT methylation as a stratification tool to select patients who could benefit from temozolomide, especially in relapsed or refractory SCLC.^69^

In addition, hypermethylation of the *RASSF1A* promoter is frequent in lung cancers, notably in NETs, and is correlated with grade, thus making a powerful tool to differentiate high‐grade NETs from non‐small cell lung carcinoma (NSCLC).^70^

In conclusion, defects in DNA methylation in lung NETs provide promising diagnostic and prognostic biomarker candidates, though some still require prospective clinical validation.

### 3.2 Histone modifications: Mechanisms, regulation and implications in cancer

**FIGURE 2:**
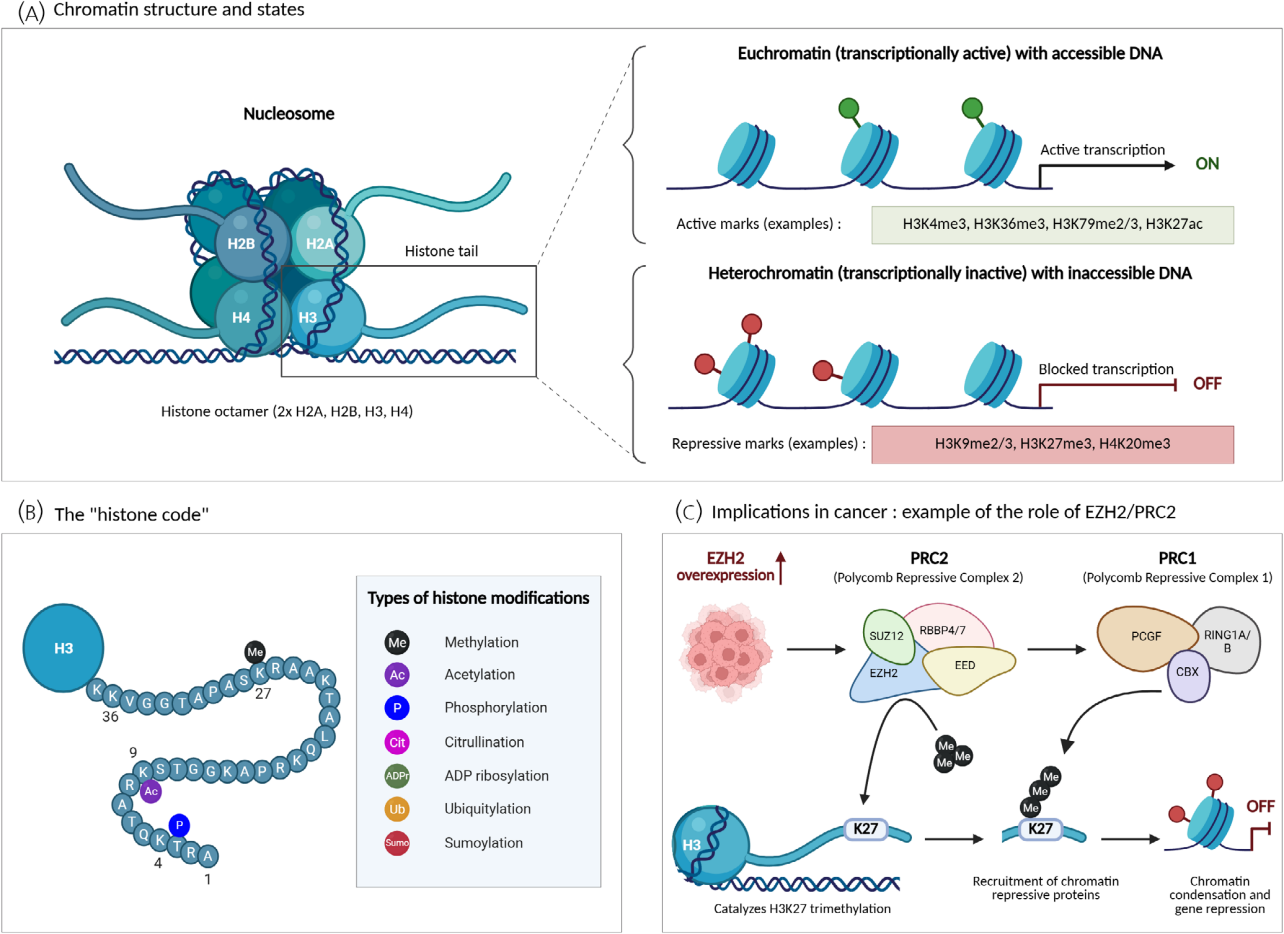
Epigenetic regulation of chromatin in cancer. (A) Nucleosomes organize DNA into euchromatin (transcriptionally active) and heterochromatin (transcriptionally repressive) states through specific histone modifications. (B) Histone post‐translational modifications, such as methylation and acetylation, modulate chromatin accessibility and gene expression. (C) An example of cancer‐associated epigenetic dysregulation. The overexpression of EZH2, a catalytic subunit of the PRC2 complex, leads to increased levels of the H3K27me3 repressive mark. This modification is recognized by the PRC1 complex, promoting chromatin compaction and gene silencing.

DNA is negatively charged and wraps around positively charged histone proteins, forming octamers composed of two copies of histones H2A, H2B, H3, and H4. This structure forms the nucleosome, which is the basic unit of chromatin.^71^ Chromatin can be transcriptionally active or inactive; a decondensed state, known as euchromatin, allows for gene transcription, whereas a condensed state, known as heterochromatin, prevents transcription. Histones can undergo post‐translational modifications, as they possess free histone tails that can be chemically modified.^72^ These modifications regulate access to DNA and can lead to either chromatin condensation or relaxation. Specific modifications to the amino termini of histones can promote synergistic or antagonistic interactions with chromatin‐associated proteins, thereby determining dynamic transitions between transcriptionally active or silent chromatin states. This concept is referred to as the ‘histone code’, due to the various possible combinations of histone modifications^73^ (Figure 2). One possible modification is histone methylation, which occurs on the side chains of lysines or arginines by histone methyltransferases (HMTs). This can either activate or repress transcription, depending on the position and state of the modification.^74^ Enzymes called histone demethylases (HDMs) can then remove methyl groups from histones. Lysines can be mono‐, di‐, or tri‐methylated, while arginines can be mono‐methylated or di‐methylated in either a symmetric or asymmetric manner.^74^ For example, methylations on H3K4, H3K36, and H3K79 are associated with active transcription, while those on H3K9, H3K27, and H4K20 are linked to non‐active transcription and repressed chromatin. These modifications also interact with other histone modifications and DNA methylation to regulate gene expression.^75^ Methylation is not the only histone modification; other enzymes can add marks such as acetylation, phosphorylation, deamination (citrullination), ADP ribosylation, ubiquitylation, and sumoylation.^74^ In humans, histone modifications can be related to both the initiation and progression of cancer. For instance, strong gene repression due to the overexpression of the enhancer of zeste homolog 2 (EZH2) protein has been associated with the progression of several solid tumours.^74^ The EZH2 protein is a member of the Polycomb Repressive Complex 2 (PRC2), which plays a key role in transcriptional repression.

#### 3.2.1 Histone modifications in siNETs

SiNET cells exhibit specific histone modification signatures distinguishing them from normal neuroendocrine cells. Notably, it has been shown that the expression of the enzyme EZH2 is high in primary tumours and metastases, albeit negligible in normal neuroendocrine cells of the small intestine.^76^ Inhibition of EZH2 using pharmacological agents such as CPI‐1205 and metformin in CNDT2.5 and GOT‐1 neuroendocrine cell lines was reported to reduce tumour cell viability and promote apoptosis. In a murine xenograft model using CNDT2.5, EZH2 knockout also inhibited tumour progression,^76^ linking this histone modifier to siNET proliferation and metastasis. Nevertheless, histone studies of siNETs are currently few and far between and largely conducted on small cohorts.

##### Histone modifications as epigenetic biomarkers in siNETs

In siNETs, histone methylation biomarkers are not frequent or clinically established. The EZH2 subunit of PRC2 catalyses H3K27 methylation and is overexpressed in siNETs. Though this subunit is druggable, it is currently used as a therapeutic target, not as an established biomarker.^76^, ^77^ Hence, histone modifications have so far not led to reliable or clinically validated biomarkers in siNETs.

#### 3.2.2 Histone modifications in pNETs

Chromatin remodelling associated gene mutations are quite common in pancreatic neuroendocrine tumours (pNETs). Exome sequencing of sporadic pNETs revealed several somatic inactivating mutations in genes involved in chromatin remodelling. *MEN‐1*, which encodes menin, a component of the histone H3K4 methyltransferase complex, was the most frequently altered gene, representing 44% of pNET modifications. In addition, nearly half of pNETs harbour mutations in either one of the two subunits of *DAXX* or *ATRX*, which encode subunits of a transcriptional and chromatin remodelling complex. *DAXX* is somatically mutated in just over 40% of the patients with pNETs.^15^, ^16^ Clinically, mutations in *MEN‐1* and *DAXX/ATRX* are associated with a better prognosis, highlighting the central role of epigenetic dysregulation in pNETs.^78^, ^79^ Moreover, in *MEN‐1*‐related pNET cells, a complete loss of menin is sufficient for tumour initiation. Somatic biallelic loss of wild‐type menin in pNETs is thought to disrupt epigenetic control of gene expression through histone modifications and DNA hypermethylation, thus promoting MEN‐1‐associated pNET tumorigenesis.^64^

pNETs also exhibit high levels of expression of multiple histone deacetylase (HDAC) subtypes compared to normal endocrine tissue, and HDAC inhibition using panobinostat was reported to induce cell‐cycle arrest, apoptosis and a redifferentiation in pNET models.^80^ Furthermore, a broad screening of >150 epigenetic inhibitors in NET lines (including pNET cell lines) revealed that compounds targeting HDACs, histone demethylases (KDMs), histone methyltransferase and bromodomain ‘readers’ markedly reduced pNET cell viability and increased apoptosis, supporting a functional dependence on the histone‐modifying machinery.^81^ Moreover, the histone methyltransferase NSD3, which catalyses H3K36me2, acts as an oncogenic driver in pNET models, and its loss reduces proliferation and H3K36me2, whereas a hyperactive mutant accelerates tumour growth.^82^

Other histone tags have also been identified, such as H3 glycation. This modification seems to play a role in cancer development and may present an interesting link with neuroendocrine tumours, such as insulinoma, with high glucose uptake due to excessive insulin production by pNETs.^83^, ^84^

In addition, in pancreatic neuroendocrine neoplasms (pNENs) and in neuroendocrine prostate cancer (NEPC), it was shown that EZH2, a PRC2 catalyst, deposits the H3K27me3 mark, leading to the silencing of tumour suppressor genes and genes involved in the cellular differentiation of cancer cells.^85^, ^86^ As a result, cancer cells remain in a proliferative and undifferentiated state. The genes repressed by the Polycomb complexes in NEPC are associated with a neuroendocrine‐associated repression signature (NEARS), which is an indicator of poor prognosis and a transition to a neuroendocrine phenotype.^85^

Overall, these findings highlight the epigenetic alterations, particularly histone modifications and dysregulation of chromatin remodelling complexes, that play a central role in the initiation and progression of pNETs, while also representing promising therapeutic targets.

##### Histone modifications as epigenetic biomarkers in pNETs

Evidence from pNET studies indicates that chromatin‐modifying proteins, notably DAXX, hold prognostic value: loss of DAXX expression consistently associates with shorter survival and more aggressive tumour progression.^62^

Interestingly, the most frequently altered genes, *MEN‐1*, *DAXX* and *ATRX* are chromatin modifiers that regulate histone methylation, such as the protein encoded by ATRX and DAXX, and are required to load H3.3 at telomeres.^79^

Hence, in pNETs, histone methylation is implicated in *MEN‐1/DAXX/ATRX*‐related chromatin regulation, but specific histone methylation biomarkers are currently not clinically established or readily available.

#### 3.2.3 Histone modifications in lung neuroendocrine tumours

Large scale sequencing of 148 lung NETs (typical/atypical carcinoids, small cell lung carcinoma (LCNEC), SCLC) revealed mutations in chromatin‐remodelling genes (including histone modifiers and SWI‐SNF components) in ~45% of carcinoids and 55% of carcinomas, the highest mutation rate among gene groups, suggesting a central role in lung NETs pathogenesis.^65^ Consistent with this finding, broader NET overviews report that lung NETs frequently harbour mutations in genes encoding histone covalent modifiers and SWI/SNF complex members, such as *MEN‐1*, *EZH1*, *CBX6*, *KDM4A*, *PHF8*, *JMJC1C*, *SETD1B*, *NSD1*, *HDAC5*, *ARID1A*, *SMARCA1/2/4*, in about 40%–80% of carcinoids (3). In line with this, multiple clinical trials in NETs, including lung NETs, are exploring inhibitors of histone modifiers and epigenetic pathways as potential treatments, reflecting the current interest in histone dysregulation.^87^

Thus, numerous studies have linked neuroendocrine tumours to epigenetic modifications, specifically DNA methylation and histone modifications.

##### Histone modifications as epigenetic biomarkers in lung NETs

In lung NETs, a study of 32 lung NETs (typical/atypical carcinoid, large cell neuroendocrine lung carcinoma or LCNEC, SCLC) revealed progressive loss of H4K16 acetylation and H4K20 trimethylation in low to high grade tumours, reflecting their differentiation level and proliferative activity. In addition, both marks were inversely correlated with Ki‐67 staining, suggesting that these histone H4 modifications mirror grade and proliferative activity and may serve as tumour biomarkers.^88^

In conclusion, altered histone methylation is present and correlated with tumour grade in lung NETs. However, histone methylation biomarkers are not yet common or clinically established. Of note, most validated epigenetic biomarkers in lung NETs remain DNA‐methylation based.

#### 3.2.4 Serotonin‐driven epigenetic control of NETs metastasis

Serotonin is a neurotransmitter with pleiotropic function in human physiology, from cerebral processes to digestive motility, and its actions are mainly mediated following its binding to its 5‐hydroxytryptamine receptor (5‐HTR). An excessive serotonin production can be observed in some NET locations, essentially small intestine and lung NETs, resulting in symptoms that can be life‐threatening such as diarrhoea, flushes, carcinoid heart disease, and bronchospasms. Less than 10 years ago, it was demonstrated that serotonin could also be linked to H3 histone via transglutaminase 2 (TGM2), allowing the enhancement of TFIID binding to H3K4me3, resulting in gene expression modulation.^89^ This translational histone modification is involved in ependymoma development, modulating the expression of several developmental transcription factors. Thus, pharmacologically targeting histone serotonylation is of therapeutic interest.^90^ Recently, using a neuroendocrine prostate cancer model, some authors highlighted that histone serotonylation participated in liver metastasis development, triggering histone condensation in polynuclear neutrophils and participating in the development of neutrophil extracellular traps in the liver, all of which converge to promote metastasis development.^91^ Interestingly, targeting histone serotonylation via TGM2 or indirectly by inhibiting the 5‐HT transporter SERT with fluoxetine, an approved antidepressant molecule routinely used worldwide, could limit neutrophil extracellular traps and liver neuroendocrine tumour metastasis.

Collectively, histone modifications play a central role in regulating gene expression and chromatin status, influencing the development and progression of NETs. Alterations in enzymes such as *EZH2*, *HDACs*, *MEN‐1*, *DAXX*, *ATRX*, and emerging marks like histone serotonylation drive tumour growth, metastasis, and resistance to therapy. These insights highlight histone regulation as both a key mechanism of NET pathogenesis and a promising target for novel epigenetic therapies.

### 3.3 Role of non‐coding RNAs in gene expression regulation and cancer

**FIGURE 3:**
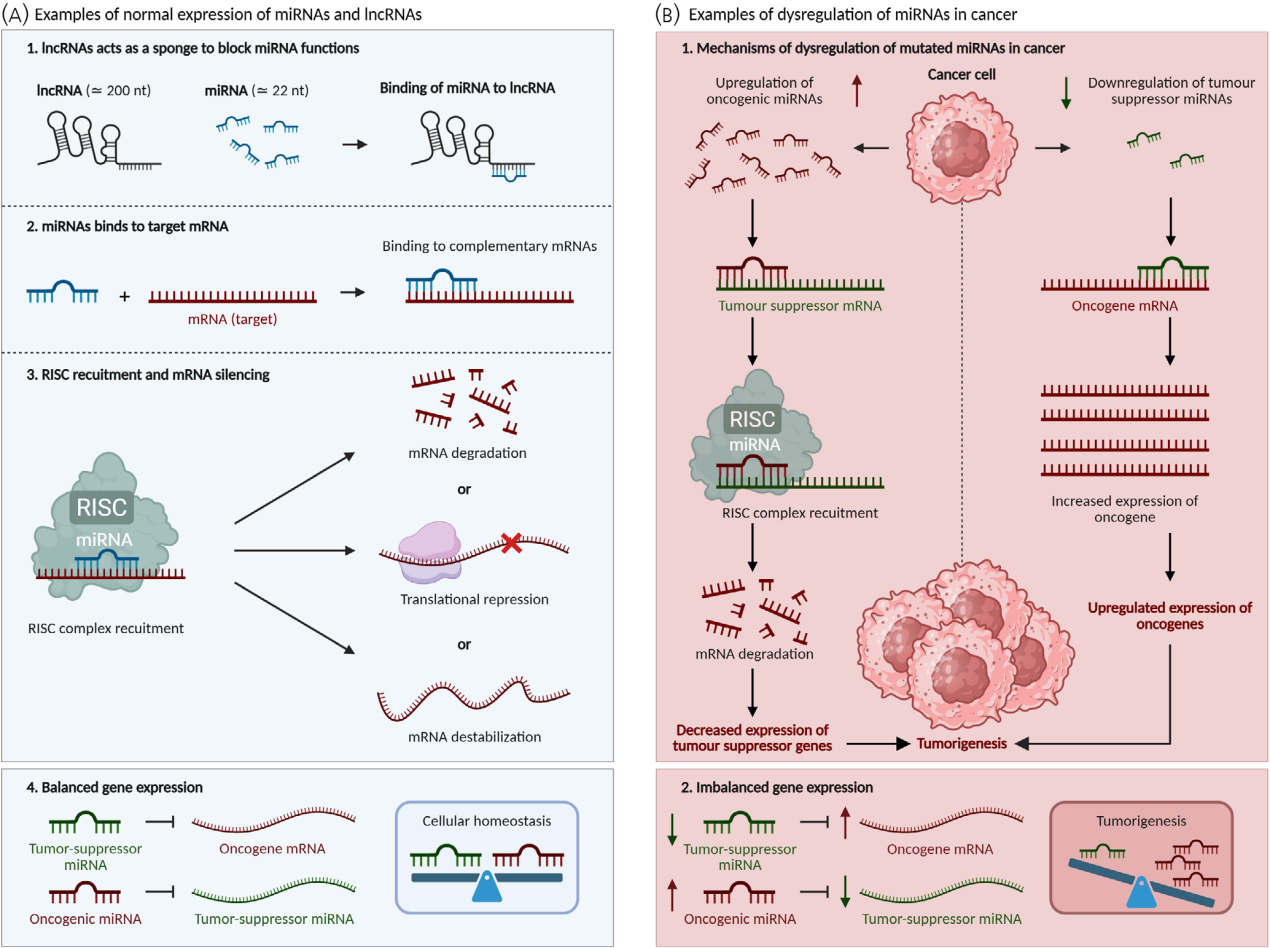
Role of non‐coding RNAs in gene expression regulation and cancer. (A) Mechanisms of non‐coding RNA‐mediated gene regulation. lncRNAs can act as molecular sponges for miRNAs, preventing their interaction with target mRNAs. miRNAs regulate gene expression through recruitment of the RISC complex, leading to mRNA degradation, translational repression, or mRNA destabilization. (B) Dysregulation of miRNAs in cancer. Upregulation of oncogenic miRNAs and downregulation of tumour suppressor miRNAs promote oncogene expression, loss of tumour suppressor activity, and tumorigenesis.

Non‐coding RNA sequences have also been shown to play a role in regulating gene expression. Most of the human genome is transcribed into RNA that does not encode proteins. Indeed, 75% of the human genome is transcribed into RNA, and only 3% of that is subsequently translated into proteins.^92^ These non‐coding RNAs (ncRNAs) are classified into different categories based on their length, shape, and localization, and play crucial roles in regulating the initiation and progression of various cancers by acting as oncogenes or tumour suppressors.^93^ Among them are long non‐coding RNAs (lncRNAs) and circular RNAs (circRNAs), both measuring over 200 nucleotides, and circRNAs can be considered a subclass of lncRNAs. lncRNAs are linear, while circRNAs are circular, and both are implicated in the epigenetic modulation of chromatin.^94^ It was shown that somatic/germline alterations of lncRNAs can drive tumour initiation and progression.^95^ Among ncRNAs, microRNAs (miRNAs) are small RNAs of about 22 nucleotides that are complementary to a sequence of mRNA to which they can bind. The binding of miRNA to mRNA leads to the destruction of the mRNA molecule by the RNA‐induced silencing complex (RISC)^96^ (Figure 3).

Moreover, miRNAs can be ‘sponged’ by lncRNAs, as they can recognize the sequence of the long non‐coding RNA and bind to it. Finally, PIWI‐interacting RNAs (piRNAs) are 23 to 32 nucleotides in length and are involved in the epigenetic regulation of chromatin by binding to proteins of the PIWI family.^97^ In animal germ cells, piRNAs protect the genome by silencing transposable elements.^97^ A dysregulation of miRNAs has also been reported in NETs, such as pNETs, siNETs, or in pulmonary carcinoids.^98^

#### 3.3.1 Non‐coding RNAs in siNETs

siNETs are among the best‐characterized NET subtypes for miRNA dysregulation. The analysis of a panel of 95 miRNA expression profiles, between primary ileal NETs and metastatic tumours, revealed a decrease in some miRNAs, such as 133a, ‐145, ‐146, ‐222, and ‐10b and an increase in others, including ‐183, ‐488, ‐19a and ‐19b, in metastases compared to the primary tumour.^99^ In addition, a global miRNA profiling of well‐differentiated siNETs (24 tumours with multiple stages) identified a distinct tumour miRNA signature with 9 miRNAs consistently dysregulated during progression, 5 upregulated (miR‐96, ‐182, ‐183, 196a, ‐200a) and 4 downregulated (miR‐31, ‐129‐5p, ‐133a and ‐215).^99^

Some of these miRNAs play key physiological roles and their dysregulation is associated with many pathologies. For example, mirRNA‐133a is located on chromosome 18 and is involved in muscle differentiation and proliferation. This miRNA is frequently down‐regulated in hypertrophic muscle tissues, in squamous carcinomas of the tongue, in hepatocellular and lung carcinomas, in neutrophils in myeloproliferative disorders, and potentially in the progression of primary ileal NET tumours to metastasis.^100^

Finally, a rectal NET lncRNA analysis revealed two novel transcripts (MSTRG30689.1, MSTRG341161.1) highly expressed in both siNETs and pNETs, hinting at shared lncRNA regulatory programmes across gastrointestinal NETs, even though siNET‐focused functional studies are not yet available.^101^

##### Non‐coding RNAs as epigenetic biomarkers in siNETs

Analyses of small‐bowel NET tissue have consistently revealed a distinct miRNA signature across multiple studies. Global profiling identified 39 dysregulated miRNAs in small bowel NETs compared to normal small bowel, with miR‐204‐5p, miR‐7‐5p, and miR‐375 being strongly upregulated and with miR‐1 and miR‐143‐3p being downregulated in lymph node and liver metastases compared to primary tumours. These findings expose these miRNAs as potential biomarkers that could be used in the future to better stratify and treat patients.^102^

In addition, a study on siNETs characterized 9 miRNAs with miR‐96, ‐182, ‐183, ‐196a and ‐200a being upregulated and miR‐31, ‐129‐5p, ‐133a and ‐215 being downregulated during tumour progression.^100^ Furthermore, circulating miRNAs miR‐125b‐5p, miR‐362‐5p, miR‐425‐5p, miR‐500a‐5p are upregulated and distinguish small bowel NETs patients from healthy patients and can be used to track residual/recurrent disease after surgery, indicating strong biomarker potential.^103^

Finally, a larger profiling study (42 tumours, 37 patients) showed that miRNA patterns define molecular subtypes of metastatic siNETs, correlated with grade, chromosomal gains, and proliferation, and that downregulation of miR‐375 predicts shorter overall survival. Indeed, tumour progression is associated with an upregulation of miR‐95 and miR‐210 and with a downregulation of miR‐378a‐3p in metastasis. In addition, a shorter overall survival is associated with miR‐375 downregulation in tumour metastases, indicating that miR‐375, which is highly expressed in siNETs, may serve as a prognostic biomarker.^104^

Overall, these findings support the relevance of specific miRNA expression as promising biomarkers of tumour progression in siNETs, reinforcing their relevance as diagnostic and prognostic tools.

#### 3.3.2 Non‐coding RNAs in pNETs

In pNETs, non‐coding RNAs are widely and frequently dysregulated, especially miRNAs and lncRNAs. Deep sequencing of pNETs versus healthy pancreas identified distinct miRNA‐mRNA signatures that clearly discriminate tumours from normal tissue and highlight dysregulated pathways in neuroendocrine function (miR‐7 and Reg family genes), adhesion (miR‐216 family and NLGN1, NCAM1, and CNTN1; miR‐670 and the claudins, CLDN1 and CLDN2), and metabolism (miR‐670 and BCAT1/MPST; miR‐129 and CTH).^105^

A case–control study of 7 pNETs and 19 normal pancreas profiled 84 miRNAs and found 14 miRNAs significantly dysregulated (miR‐1, miR‐133a‐3p, miR‐210‐3p, miR‐7‐5p, miR‐10a‐5p, miR‐92b‐3p, miR‐132–3p, miR‐221‐3p, miR‐29b‐3p, miR‐107, miR‐103a‐3p, let‐7b‐5p, miR‐148a‐3p, and miR‐202–3p), with a 6‐miRNA panel distinguishing pNETs from non‐tumoral tissue.^106^

Moreover, recurrent changes in miR‐21, miR‐103/107, miR‐204 and miR‐155 were shown, the first correlated with Ki‐67 and liver metastasis, reinforcing the fact that miRNA dysregulation is a consistent feature of pNET biology.^107^

Finally, in MEN‐1 non‐functional pNETs, exosomal miR‐451 and lncRNA NEAT1_1 are frequently dysregulated; NEAT1_1 levels are strongly linked to STAT3 activation in tumour tissue, supporting a functional ncRNA pathway in pNET tumorigenesis.^108^

Overall, these studies demonstrate that recurrent dysregulation of non‐coding RNAs contributes to altered neuroendocrine function, tumour progression and metastasis, further highlighting the relevance of non‐coding RNAs signatures as diagnostic biomarkers and therapeutic targets in pNETs.

##### Non‐coding RNAs as epigenetic biomarker in pNETs

Across pNET profiling studies, characteristic miRNA signatures emerge that differentiate pNET tissue from normal pancreas and from other pancreatic tumours. Indeed, disease‐associated miRNA signatures have been identified in pNETs, exhibiting distinct patterns that were correlated with tumour grade, metastasis status and Ki‐67 index. For example, an upregulation of miR‐196a is significantly associated with a decrease in overall survival of pNET patients, and miR‐21, miR‐642, and miR‐210 have been identified as prognostic markers for aggressive tumours correlated with a high Ki‐67 index, advanced stages, and metastatic spread.^106^ The expression of miR‐103 and miR‐107 with a downregulation of miR‐155 has also been shown to characterize neoplasmatic but not normal pancreatic tissue.^107^

In addition, studies reported an upregulation of miR‐7‐5p and miR‐129‐5p and a downregulation of the miR‐216 family in NETs, including pNETs compared to non‐NET controls. miR‐7‐5p in particular may influence pancreatic neuroendocrine progression as its level was shown to be 48‐fold higher in all NET cases compared to control and as its upregulation is correlated to inhibition of cell proliferation and induced apoptosis; therefore, indicating that these miRNAs can be of interest as a therapeutic and diagnostic tool in the future. Moreover, numerous differentially expressed miRNAs, with networks involving miR‐7, miR‐216, miR‐670, miR‐129, and others, were correlated with alteration in cell adhesion, metabolism, and neuroendocrine function, providing a basis for further research into using miRNAs as possible diagnostic and predictive biomarkers.^105^

Overall, miRNA panels can discriminate grade 1 versus grade 2 pNETs with ~83% sensitivity and 88% specificity, highlighting their relevance for patient stratification. Indeed, miR‐106b + miR‐130b 3p + miR‐127‐3p + miR‐129‐5p + miR‐30d‐5p were sufficient to discriminate between G1 and G2 pNETs.

Finally, broad tumour endocrine reviews have emphasized that circulating miRNAs and lncRNAs could be promising, though not yet clinically demonstrated, biomarkers for pNETs.^109^

In conclusion, non‐coding RNAs (especially miRNAs and lncRNAs) are commonly and largely altered in pNETs and are thus highly relevant as diagnostic, prognostic, and mechanistic biomarkers. However, no detection of non‐coding RNA is currently included in standard clinical management; their use mainly being confined to fundamental research and early translational studies.

#### 3.3.3 Non‐coding RNAs in lung neuroendocrine tumours

Regarding lung NETs, a profiling study of 12 lung NETs (typical/atypical carcinoid, SCLC, LCNEC) screening 763 miRNAs found 44 significantly differentially expressed miRNAs across subtypes, with 12 highly significant (*p* < .01) and several negatively (miR‐22, miR‐29a, miR‐29b, miR‐29c, miR‐367*; miR‐504, miR‐513C, miR‐1200) or positively (miR‐18a, miR‐15b*, miR‐335*, miR‐1201) correlated with grade and survival (miRNAs let‐7d; miR‐19; miR‐576‐5p; miR‐340*; miR‐1286). These miRNA signatures distinguish carcinoids from high‐grade NECs, and are associated with tumour aggressiveness, supporting a pervasive role for miRNA dysregulation in lung NET biology.^110^

In addition, upregulation of miR‐494‐3p activates Notch 1 and PI3K signalling pathways, enhancing tumour‐initiating cell proliferation, while miR‐145 was reported to inhibit it by repressing OCT4 mRNA. Downregulation of miR‐31 together with upregulation of let‐7 was shown to cooperatively induce cell cycle arrest of tumour‐initiating cells. Furthermore, STAT3 activation induced upregulation of the lncRNA HOX transcript antisense RNA, named HOTAIR, via its promoter, prompting lung tumorigenesis through the epithelia‐mesenchymal transition (EMT).^111^

Thus, the dysregulation of ncRNAs and particularly miRNAs can play a key role in cancer and NETs by promoting tumour development and progression. Altogether, these studies highlight the importance of epigenetic mechanisms in NET tumorigenesis and indicate that this field deserves further exploration in the future to improve our understanding of these rare malignancies and provide new treatment options.

##### Non‐coding RNAs as epigenetic biomarker in lung NETs

In lung NETs, evidence from Maringer et al. demonstrates that several miRNAs exhibit grade‐dependent expression patterns, with some increasing and others decreasing as tumour grade rises. In this study, members of the miR‐29 family were negatively correlated with tumour grade and were particularly important in lung NETs. These data show that miRNA dysregulation is common and subtype‐specific in lung NETs and may support diagnosis, grading, and prognosis, though this is not yet used routinely.^110^ In conclusion, non‐coding RNAs can be altered in lung NETs and show promise as biomarkers, even though they are still under research.

Taken together, these studies demonstrate that non‐coding RNAs, particularly miRNAs, can be dysregulated across siNETs, pNETs, and lung NETs. This highlights their strong potential as biomarkers, although their clinical integration remains largely investigational.

## 4 EPIGENETIC THERAPIES

### 4.1 Targeting DNA methylation

Several recent studies have explored the targeting of epigenetic mechanisms as cancer therapy. Treatments such as 5‐azacytidine and 5‐aza‐2′‐deoxycytidine inhibit DNA methylation, making them promising therapeutic agents as they silence key regulatory genes^112^, ^113^ (Figure 2). 5‐aza‐2′,2′‐difluorodeoxycytidine (NUC013) is also being investigated as a promising cancer treatment and has shown efficacy in mouse xenograft models by increasing survival in this model.^112^ This compound targets two enzymes: DNMT, which is responsible for DNA methylation, and ribonucleotide reductase (RNR), essential for deoxyribonucleotide synthesis, thus contributing to cancer cell proliferation. However, in several other malignancies, the clinical utility of DNA methylation inhibitors such as decitabine is limited by its intrinsic instability and by dose‐limiting haematologic toxicities,^114^ underscoring the need to develop next‐generation compounds that circumvent these constraints.

### 4.2 Targeting histone modifications

**FIGURE 4:**
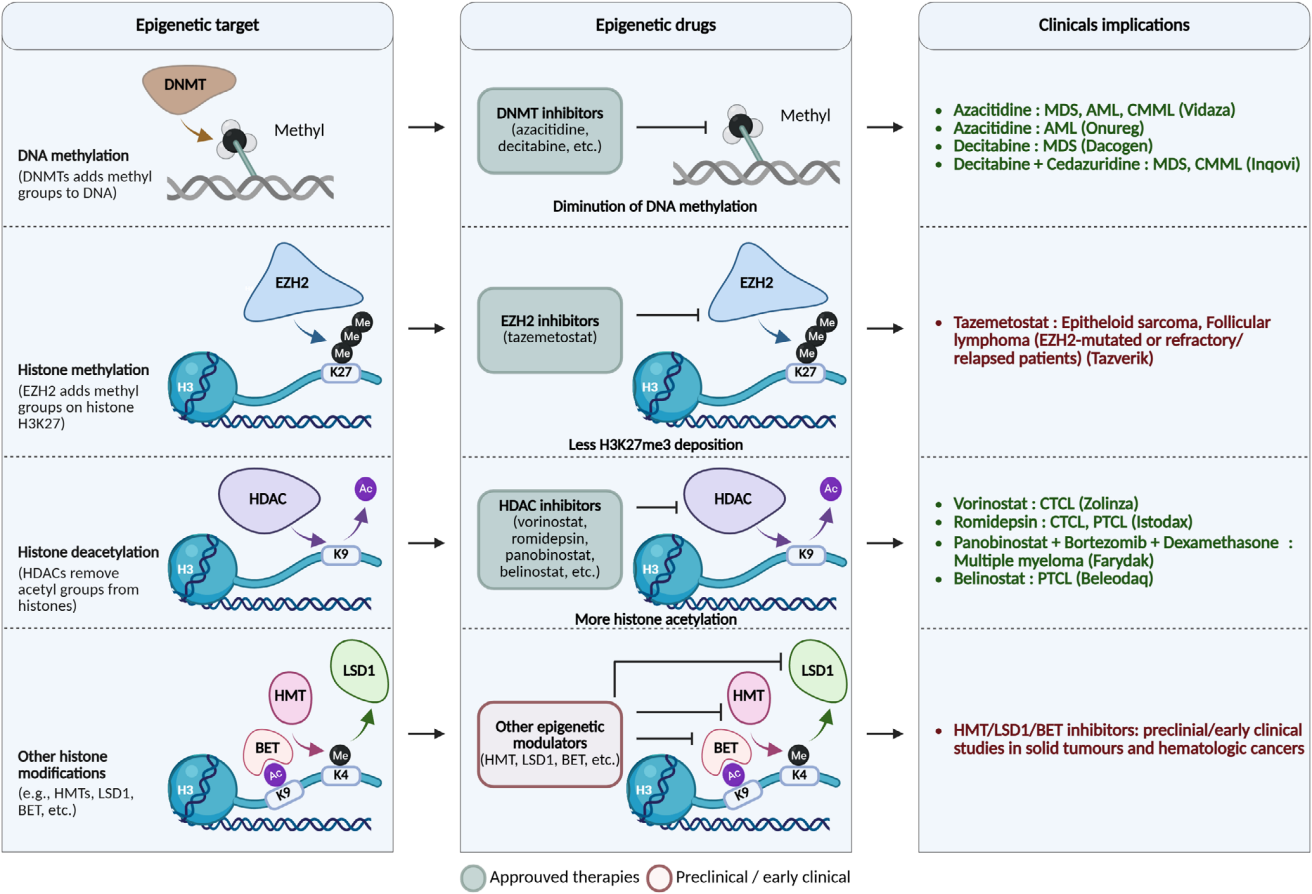
Overview of epigenetic‐targeting cancer therapies: Approved molecules and preclinical candidates. Approved therapies are shown in green, and those currently in preclinical studies are shown in red. DNMT, EZH2 and HDAC inhibitors can reverse aberrant epigenetic alterations and are clinically approved for several haematological malignancies, while other epigenetic modulators remain under clinical investigation.

Several phase II studies have explored the therapeutic potential of HDAC inhibitors in neuroendocrine tumours, although their clinical impact has remained limited (Figure 4). One of the earliest efforts evaluated valproic acid in a small cohort of eight patients with siNETs and pNETs.^115^ Despite the absence of objective responses, most evaluable patients (83%) achieved disease stabilization, with a median PFS of 13 months. Toxicity was generally manageable, with only one patient experiencing grade ≥3 adverse events, namely fatigue and hyponatremia.

Panobinostat was subsequently assessed in a broader population of 15 patients with NETs of diverse anatomical origins. Here again, no objective responses were observed, prompting early termination of the study according to its Simon two‐stage design. Median PFS reached 9.9 months, and the toxicity profile was dominated by grade ≥3 thrombocytopenia and fatigue, along with gastrointestinal symptoms such as anorexia, diarrhoea, and nausea.^116^

Another HDAC inhibitor, depsipeptide/romidepsin, was tested in a similarly heterogeneous group of 15 patients. This trial was halted prematurely due to multiple cardiac adverse events, preventing assessment of the primary endpoint and PFS. The most frequent severe toxicities included anaemia and nausea, each occurring in 13% of patients.^117^

Finally, entinostat was examined in a small cohort of four patients who had received a median of 1.5 prior treatment lines. As with previous agents, no objective responses were recorded, and the study was discontinued early by the sponsor. Median PFS was 12.5 months, and grade 3 toxicities consisted mainly of neutropenia and hypophosphatemia.^118^

Taken together, these trials illustrate the challenges of translating HDAC inhibition into meaningful clinical benefit for NET patients. While disease stabilization was occasionally observed, objective responses were consistently absent, and toxicity—particularly haematologic and cardiac—often limited treatment continuation. These findings underscore the need for more refined patient selection strategies, combination approaches, or next‐generation epigenetic modulators to fully assess the therapeutic potential of targeting chromatin regulation in NETs.

As discussed above with regards to histone modifications, EZH2 overexpression leads to gene repression and is associated with poor patient prognosis. Studies have shown that targeting EZH2 with small interfering RNA (siRNA) in metastatic prostate cancers can inhibit proliferation.^119^ Another treatment targeting EZH2 is tazemetostat, an inhibitor that has shown promising results in pNENs.^86^ A therapeutic synergy may be possible in this cancer between EZH2 inhibitors and mTOR pathway inhibitors (already used in pNENs treatment) as pNENs are driven in part by mTOR pathway activation and as EZH2 knockdown reduced viability and tumour burden in pNEN cell lines, tumoroids and mouse models.^86^ In line with EZH2, HMT inhibitors are under development or clinical use to reactivate tumour suppressor genes or repress oncogenes (90).

Other epigenetic therapies are under consideration; for example, in GEP‐NETs, histone deacetylase inhibitors (HDACi) may be used as future treatments.^80^

In an ongoing study, higher or lower doses of belinostat, a HDACi, are being tested, based on the UGT1A1 genotype in patients treated for high‐grade neuroendocrine carcinomas (NCT06406465). There are also two ongoing phase 2 studies evaluating the efficacy of the chidamide HDACi in combination with chemotherapy (platin/etoposide) or immunotherapy (sintilimab) in neuroendocrine carcinoma and high‐grade neuroendocrine neoplasms, respectively (NCT05076786 and NCT05113355) (Table 1).

**TABLE 1:** Ongoing trials evaluating epigenetic‐targeting drugs in neuroendocrine neoplasms.

Trial	Treatment	Mechanisms of action	Population	Phase	Planned enrolment	Primary endpoint
NCT06406465	Belinostat (+ platin/etoposide)	HDACi	Extrapulmonary neuroendocrine carcinoma	2	60	PK
NCT05178693	ASTX727 (cedazuridine + decitabine) (+ 177Lu‐DOTATATE)	DNMTi	Neuroendocrine tumour	1	27	SSTR2 re‐expression on PET‐CT
NCT05076786	Chidamide (+ platin/etoposide)	HDACi	Extrapulmonary neuroendocrine carcinoma	2	28	Objective response rate (ORR)
NCT05113355	Chidamide (+ sintilimab)	HDACi	High‐grade neuroendocrine neoplasm	2	23	Objective response rate (ORR)

Collectively, one could argue that the aforementioned trials are disappointing because they are all negative with no objective response observed. Nevertheless, these results should be put into perspective with the literature on NET clinical trials. Indeed, it is typical to observe low‐rate response with targeted therapy or anti‐angiogenics. In prospective phase 2–3 trials, everolimus achieved ORR ranging from 2% to 22%, while antiangiogenic agents showed ORR of 5% with sunitinib and up to 19% with cabozantinib. It was shown that ORR was probably not the best primary endpoint in early phase trials because there is no strong correlation between ORR and PFS (*r* = 0.37), a classical endpoint in recently reported NET phase 3 trials.^120^ Moreover the 12‐month PFS rate would probably be more appropriate (*r* = 0.93). When one looks at PFS data, even though this data suffers from many limitations, including the very small number of patients, results ranged from ~10 to ~13 months PFS; therefore, it seems reasonable not to abandon this therapeutic class, especially if biomarkers are discovered that could identify good candidate patients. The design of clinical trials should focus on well‐differentiated NETs, regardless of the primary location, and examine evidence of synergy with currently used drugs to test treatment combinations. Consistently, several trials are underway in multiple primitive species to test the potential synergy of drugs targeting epigenetics with chemotherapy, radiotherapy, or immunotherapy.^121^, ^122^ Their use in neuroendocrine tumours (NECs) is much more reserved at present given the lack of dedicated data.

### 4.3 Targeting non‐coding RNAs

Finally, epigenetic therapies targeting ncRNA include molecules that mimic RNAs, antisense oligonucleotides (ASOs), and small molecules specifically targeting ncRNAs.^123^ For example, miR‐34a MRX34, which is an RNA mimic, has been tested in a Phase I clinical trial in solid tumours, and MALAT1 (metastasis‐associated lung adenocarcinoma transcript 1), targeting lncRNAs, has been studied for its ability to inhibit lung cancer metastases.^124^ In summary, targeting ncRNAs appears to be a promising approach for cancer therapy.

### 4.4 Epigenetic regulation of SSTR2

Targeting SSTR2 via somatostatin analogues or peptide receptor radionuclide therapy (PRRT) is routinely conducted in the context of NETs. However, these treatments can fail, and some patients express low levels of SSTR2, especially in high‐grade NETs, leading to the development of drugs harbouring SSTR2 upregulation properties.

Several in vitro studies revealed that SSTR2 expression was linked to both DNA promoter methylation and histone acetylation (Table 2). Coherently, using a DNA methyltransferase inhibitor (DNMTi) or histone deacetylase inhibitor (HDACi), in monotherapy and/or in combination, has been shown to upregulate SSTR2 expression, objectived with somatostatin receptor PET‐CT: 5‐aza‐2′‐deoxycytidine, valproic acid, CI‐994, entinostat, LMK‐235, mocetinostat, panobinostat, vorinostat, LSD1 inhibitors.^125^, ^126^, ^127^, ^128^, ^129^, ^130^, ^131^ In preclinical models this was associated with better performances of PRRT, especially with valproic acid which also exhibits radiosensitizing properties.^132^

**TABLE 2:** Published trials evaluating epigenetic‐targeting drugs in neuroendocrine tumours.

Treatment	Mechanisms of action	Population	Phase	*n*	Primary endpoint	Median PFS	Grade ≥3 side effect	Specific side effect	References
Depsipeptide	HDACi	NET, various location	2	15	ORR: not determined	Not determined	Anaemia (13%), nausea (13%)	Grade 2 ST depression/T‐inversion (33%), Grade 2 QTc prolongation (20%), Ventricular tachycardia (13%)	100
Valproic Acid	HDACi	NET, small intestine and pancreas	2	8	Response rate: 0%	13 months	Fatigue and hyponatremia: 1 patient		98
Panobinostat	HDACi	NET, various location	2	15	ORR: 0%	9.9 months	Thrombocytopenia (27%), fatigue (27%), anorexia (13%), diarrhoea (13%), nausea (13%)		99
Entinostat	HDACi	NET, various location	2	4	ORR: 0%	12.5 months	Neutropenia: 2 patients, hypophosphatemia: 1 patient		101

To date there are no clinical data published on NET patients testing a combination of conventional drugs targeting SSTR2 (i.e., cold somatostatin analogues or PRRT). However, one study is ongoing combining Lu‐DOTATATE with DNMT inhibitors ASTX727 (cedazuridine + decitabine) (NCT05178693).^133^

Conventional treatments for NET patients may lead to drug resistance.^10^ When this occurs, a change in medical management may be considered with, for example, a combination of treatments. Therefore, the use of epigenetic therapies could be a new therapeutic strategy in the future. However, it should also be noted that resistance mechanisms to therapies targeting methylation may also exist. Indeed, mutations in HMTs, for example, can occur, leading to the activation of compensatory pathways (allowing cells to maintain their proliferation), as well as an increase in cellular plasticity (to escape therapeutic pressure).^44^ Another resistance phenomenon can be found in adult patients with T‐cell leukaemia/lymphoma who have been treated with a dual inhibitor of EZH1‐EZH2, valemetostat. Indeed, a clonal selection of the PRC2 gene mutation or a hypermethylation of the DNA may be responsible for resistance to treatment. In response to this, therapeutic combinations, the identification of biomarkers, and the development of more specific inhibitors could improve patient treatment approaches.^44^

## 5 PERSPECTIVES AND FUTURE DIRECTIONS

As the field of neuroendocrine tumour (NET) research continues to expand, the epigenetic perspective is offering a more nuanced understanding of how these tumours emerge, adapt, and resist therapy. What once appeared as biologically ‘quiet’ cancers are now recognized as epigenetically dynamic diseases, shaped by chromatin remodelling, DNA methylation, and microenvironmental cues. Recent work underscores how deeply these mechanisms influence NET identity and behaviour, and it also highlights how much remains to be explored.^134^

A major priority moving forward is to comprehensively capture the full extent of epigenetic heterogeneity across NET subtypes. Current datasets, although increasingly sophisticated, still fail to comprehensively characterize rare and advanced disease stages. Large, collaborative efforts integrating DNA methylation, histone modifications, chromatin accessibility, and non‐coding RNA profiling will be essential for building a more complete map of NET biology. Furthermore, spatial multi‐omics technologies, which map cellular and molecular heterogeneity directly onto tissue architecture, are reshaping how we view the tumour microenvironment and revealing new possibilities for precision treatment.^135^

Equally important is the shift from descriptive to functional epigenetics. Several methylation signatures and chromatin regulators have been identified in NETs, but their causal roles in lineage plasticity, metastatic spread, and treatment resistance remain insufficiently understood. Studies in other tumour types show how epigenetic dysregulation can drive therapy resistance and reshape interactions between the tumour and the microenvironment, suggesting that similar mechanisms may be involved in NET tumorigenesis.^134^ Mechanistic work using organoids, co‐culture systems, and in vivo models will be crucial for uncovering actionable vulnerabilities.

Clinically, epigenetic biomarkers could help improve NETs diagnosis and better predict disease progression and treatment response. DNA methylation profiling has already demonstrated its power in classifying pituitary NETs into clinically meaningful subgroups, offering a glimpse of what could be achieved across the broader NET pathologies.^136^ Beyond classification, methylation‐based assays hold promise for early detection, minimal residual disease monitoring, and distinguishing indolent from aggressive disease. Yet, as highlighted in recent reviews, the translation of methylation biomarkers into routine practice still requires rigorous validation and standardization.^137^

Therapeutically, the next decade will likely witness an expansion of mechanistically targeted epigenetic strategies. While classical DNMT and HDAC inhibitors have shown limited efficacy, emerging strategies targeting chromatin remodelers, enhancer rewiring, or transcriptional dependencies may offer more precision. Combination therapies, pairing epigenetic agents with immunotherapy, targeted drugs, or radionuclide therapy, represent an especially promising direction, supported by broader evidence that epigenetic modulation can help overcome therapeutic resistance.^138^

Finally, one of the most exciting frontiers lies in integrating epigenetic data with spatial and single‐cell technologies. Spatial multi‐omics is beginning to reveal how NET cells interact with their microenvironment and how these interactions shape tumour evolution. Such integrative approaches could ultimately enable predictive models of NET progression and guide more durable therapeutic strategies.^139^, ^140^

In conclusion, NETs represent a distinct group of malignancies whose biological behaviour cannot be fully explained by genetic alterations alone. The growing body of evidence summarized in this review underscores the central role of epigenetic regulation in NET development, progression, and cellular plasticity, offering important insights into their pathogenesis. Advances in our understanding of epigenetic mechanisms have already contributed to improving diagnostic and prognostic strategies and hold significant promise for the development of novel therapeutic approaches. By integrating current clinical knowledge with emerging epigenetic findings, this review provides a framework that supports ongoing research efforts and fosters a deeper understanding of NET biology, ultimately aiming to improve patient management and outcomes in these rare and complex malignancies.

## AUTHOR CONTRIBUTIONS

**Victoria Jacquot:** Conceptualization; writing – original draft; writing – review and editing. **Maria Ouzounova:** Conceptualization; writing – review and editing; writing – original draft; supervision. **Benjamin Gibert:** Writing – review and editing. **Thomas Walter:** Writing – review and editing. **Benjamin Chevalier:** Conceptualization; writing – review and editing; writing – original draft. 

## CONFLICT OF INTEREST STATEMENT

The authors declare no conflicts of interest.

## Data Availability

Data sharing not applicable to this article as no datasets were generated or analysed during the current study.
